# Protein phosphatase 2A methylation state impacts α-synucleinopathy in mouse models

**DOI:** 10.1038/s41420-026-03045-7

**Published:** 2026-03-24

**Authors:** Santhosh Maddila, Kambiz Hassanzadeh, Jun Liu, Jie Zhang, Faheem Ullah, Russell E. Nichols, M. Maral Mouradian

**Affiliations:** 1 Department of Neurology and Robert Wood Johnson Medical School, Institute for Neurological Therapeutics, Rutgers Biomedical and Health Sciences, Piscataway, NJ USA; 2 Department of Pathology and Cell Biology, and The Taub Institute for Research on Alzheimer’s Disease and the Aging Brain, Columbia University, New York, NY USA

**Keywords:** Parkinson's disease, Parkinson's disease

## Abstract

The accumulation of aggregated alpha-Synuclein (α-Syn) in Lewy bodies and Lewy neurites is a hallmark of Parkinson’s disease (PD) and Dementia with Lewy Bodies (DLB), and phosphorylation of α-Syn at Ser129 is a key pathological marker in synucleinopathies. The heterotrimeric enzyme protein phosphatase 2A (PP2A), and specifically its B55α containing isoform, which dephosphorylates phospho-S129-α-Syn, is regulated through methylation of its catalytic C subunit, a process that is controlled by the opposing activities of leucine carboxyl methyltransferase 1 (LCMT-1) and protein phosphatase methylesterase 1 (PME-1). Postmortem studies show decreased LCMT-1 and increased PME-1 levels in PD and DLB brains, leading to reduced PP2A activity and α-Syn hyperphosphorylation. To investigate the pathophysiological relevance of this regulatory axis, we employed genetically modified mice in two models of synucleinopathy, transgenic animals and intrastriatal α-Syn preformed fibrils (PFF) injections. A battery of behavioral tests was conducted to assess motor and cognitive function, followed by brain analyses quantifying phosphorylated α-Syn aggregates, neuronal toxicity, and neuroinflammatory responses, thereby evaluating how modulation of this axis influences α-Syn pathology. Overexpression of PME-1 in forebrain neurons exacerbated α-Syn pathology, characterized by increased Ser129 phosphorylation and aggregation, as well as neurodegeneration and neuroinflammation, accompanied by significant motor impairments. These effects were observed both in transgenic mice co-expressing PME-1 and human α-Syn at 9 months of age, and in PME-1 overexpressing mice six months after intrastriatal injection of α-Syn PFF. In contrast, LCMT-1 overexpression reduced α-Syn phosphorylation and aggregation, and provided robust neuroprotection, leading to improved motor outcomes in both synucleinopathy models. These findings underscore the critical role of PP2A methylation dynamics in regulating α-Syn toxicity. Accordingly, targeting the PP2A methylation machinery represents a promising therapeutic strategy to mitigate α-Syn-induced neurodegeneration and slow the progression of synucleinopathies.

## Introduction

α-Synuclein (α-Syn) accumulates in Lewy bodies and Lewy neurites, the hallmark pathological features of synucleinopathies, including Parkinson’s disease (PD) and Dementia with Lewy Bodies (DLB) [1]. A well-recognized and prevalent marker of pathological α-Syn in these disorders is its hyperphosphorylation at Serine 129 (pS129). This post-translational modification (PTM) promotes α-Syn aggregation and fibrillization in vitro [2, 3] and transforms it into a structurally distinct and functionally more toxic strain. Conversely, reducing this PTM may mitigate toxic α-Syn strain formation [4]. Therefore, identifying factors driving this process is crucial for understanding the molecular mechanisms underlying synucleinopathies and could open new avenues for developing disease-modifying therapies.

The phosphorylation level of proteins, including α-Syn, is maintained through a dynamic balance between kinase-mediated phosphorylation and phosphatase-mediated dephosphorylation [5]. Considering that multiple kinases contribute to α-Syn phosphorylation [5], targeting a single kinase with an inhibitor is likely ineffective [6]. An alternative and potentially more useful approach is to focus on the phosphatase responsible for dephosphorylating pS129-α-Syn [7]. Through a series of studies, protein phosphatase 2A (PP2A) has been identified as the major enzyme involved in α-Syn dephosphorylation, with evidence suggesting an inverse correlation between PP2A activity and α-Syn phosphorylation and aggregation [8–11].

PP2A is a highly conserved family of enzymes expressed ubiquitously, including in the brain, that play the crucial role as serine/threonine phosphatases [12, 13]. They function predominantly as trimeric holoenzymes composed of a catalytic C subunit (PP2Ac) and a scaffolding A subunit (PP2Aa/PR65), assembled with one of multiple regulatory B subunits (PP2Ab) [14]. The regulatory subunits of PP2A are classified into four subfamilies (B, B′, B″ and B‴), which confer substrate specificity by influencing holoenzyme composition and function [15]. The methylation of the catalytic C subunit at Leu309 by leucine carboxyl methyltransferase 1 (LCMT-1) enhances its affinity for regulatory B subunits (B55), thereby increasing PP2A holoenzyme activity [14]. In contrast, protein phosphatase methylesterase-1 (PME-1) functions opposite to LCMT-1 by demethylating PP2Ac [16], leading to the removal of Mn²⁺ from the catalytic site, thereby inhibiting its activity [17]. In addition to its demethylation function, PME-1 contributes to PP2A regulation by stabilizing the complex and maintaining a fraction of PP2Ac in an inactive state [18]. The PP2A isoform containing the B55α subunit is the primary mediator of pS129-α-Syn dephosphorylation [9] and is, thus, a significant factor in regulating the phosphorylation state and aggregation potential of α-Syn.

Postmortem analyses of PD and DLB-affected brains have shown significant dysregulation of PP2A and its methylation-regulating enzymes [9, 19]. Immunohistochemical studies have revealed a reduction of LCMT-1 levels in the substantia nigra (SN) and frontal cortex of PD and DLB-affected cases, while PME-1 levels are increased in the SN of PD brains compared to age-matched controls. These changes correlate with decreased methylated / demethylated PP2A ratio, while total PP2A levels are no different from controls, suggesting dysregulated PP2A methylation dynamics in these neurodegenerative conditions [20]. In addition, we previously demonstrated that pharmacological activation of PP2A using a PME-1 inhibitor enhances α-Syn dephosphorylation both in vitro and in vivo, resulting in reduced α-Syn phosphorylation levels and aggregation, diminished neuropathological changes and improved behavioral performance in a transgenic mouse model of synucleinopathy [9].

Given the critical role of PP2A methylation in regulating its catalytic activity and its potential contribution to synucleinopathies, the present study aimed to investigate the direct role of key enzymes involved in PP2A methylation in α-Syn pathology. To address this, we utilized two mouse models of synucleinopathies. First, we employed genetically modified transgenic mice expressing both α-Syn and PME-1 or α-Syn and LCMT-1, and compared these lines with α-Syn-only transgenic and wild-type (WT) mice. Second, we used the α-Syn preformed fibril (PFF) model, in which WT mice or transgenic mice overexpressing PME-1 or LCMT-1 were subjected to PFF injections into the striatum to induce synucleinopathy. Behavioral assessments were performed to evaluate disease phenotypes in both models, followed by immunohistochemical analyses of the brain to assess molecular and pathological changes.

## Results

### PP2A methylation modulates pS129-α-Syn aggregate formation in α-Syn transgenic mice

**Fig. 1:**
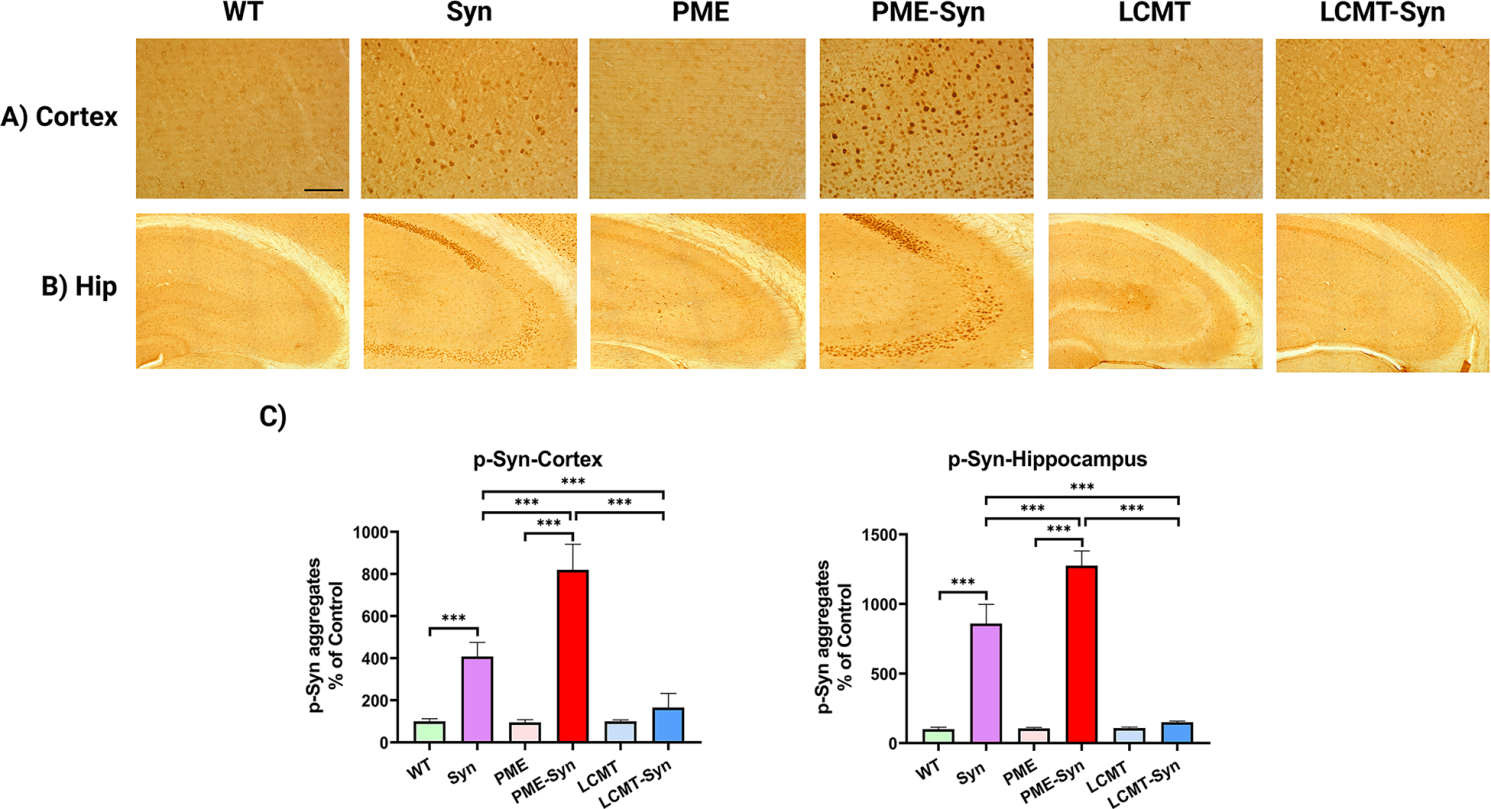
PP2A methylation reduces p-α-syn aggregate formation. Representative IHC images showing p-α-Syn aggregates in **A** cortex and **B** hippocampus. The scale bar represents 100 µm. **C** Quantification of staining intensity. Bar graphs display means ± SEM (*n* = 5 mice per group). Statistical significance was assessed using one-way ANOVA followed by Tukey’s post hoc test. ***p < 0.001. Genotypes: WT (wild-type), Syn (α-Syn transgenic), PME (PME-1 overexpressing), PME-Syn (PME-1 and α-Syn Triple transgenic), LCMT (LCMT-1 overexpressing), and LCMT-Syn (LCMT-1 and α-Syn Triple transgenic).

To investigate the role of PP2A methylation in α-Syn–induced pathology, we generated triple-transgenic mice by crossing α-Syn transgenic mice (under the control of the pan-neuronal Thy1 promoter) [21, 22] with either PME-1/CaMKIIα mice (hereto forth referred to as PME-1 mice), which overexpress PME-1 in the forebrain driven by a synthetic transactivator that is under the control of the calcium calmodulin kinase IIα (CaMKIIα) gene promoter and exhibit PP2A demethylation, or LCMT-1/CaMKIIα mice (hereto forth referred to as LCMT-1 mice), which similarly overexpress LCMT-1 in the forebrain [23]. At 9 months of age, after completing behavioral studies, mice were euthanized and their brains collected for histopathological analysis. Immunohistochemical staining for pS129-α-Syn revealed a significant increase in aggregates in the cortex and hippocampus of α-Syn transgenic mice compared to wild-type controls, as is well established in this model [9, 21, 22]. Notably, PME-1/α-Syn triple-transgenic mice exhibited further increase in pS129-α-Syn aggregation, suggesting that PME-1–mediated PP2A demethylation exacerbates α-Syn pathology. In contrast, LCMT-1/α-Syn mice showed a marked reduction in pS129-α-Syn aggregates relative to both α-Syn and PME-1/α-Syn mice, which was comparable to levels seen in WT littermate mice and supports a protective role for PP2A methylation in limiting α-Syn aggregate formation (Fig. 1).

### PP2A methylation mitigates α-Syn-induced neuronal damage

**Fig. 2:**
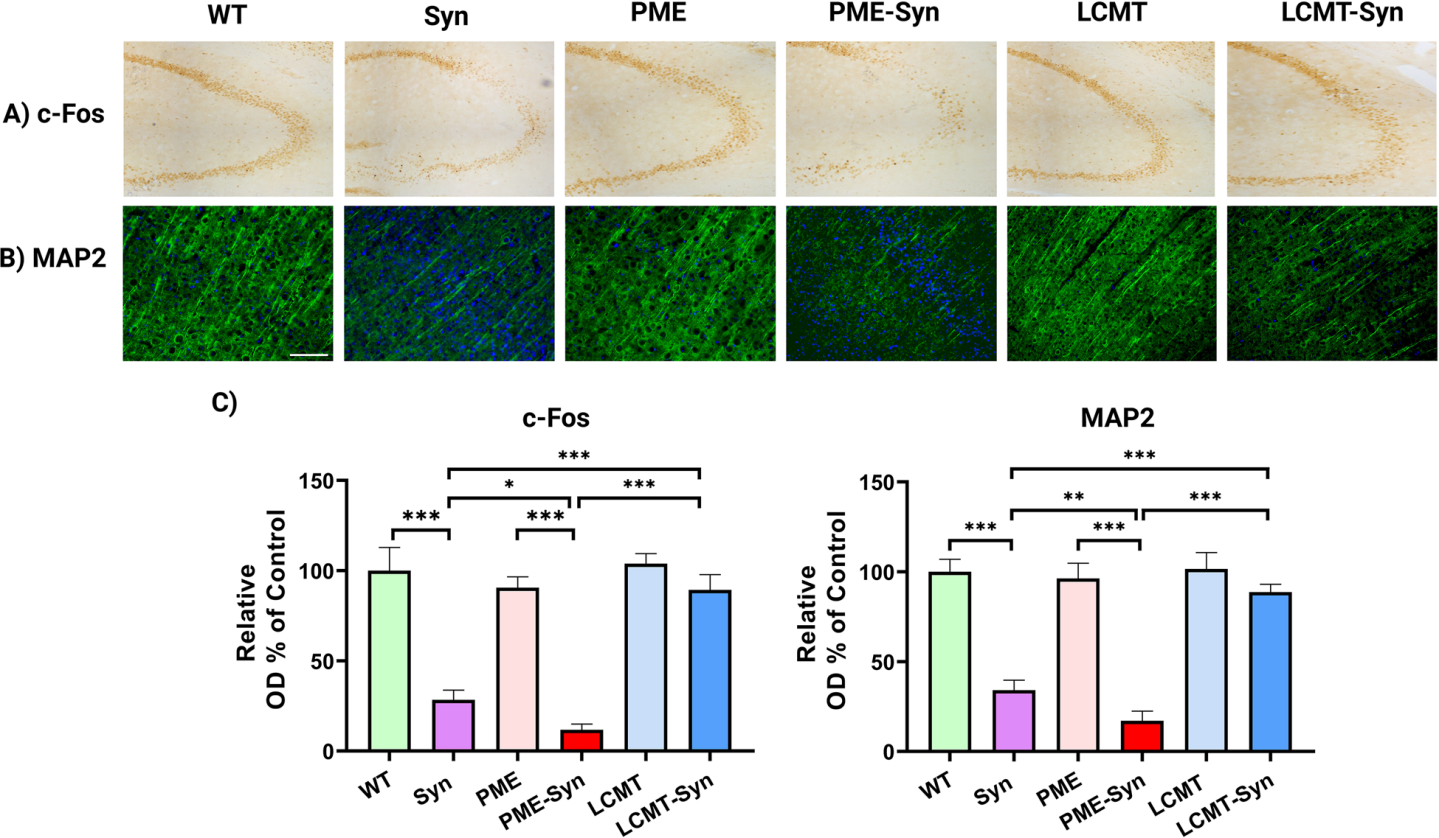
PP2A methylation influences neuronal function and integrity. Representative immunohistochemical images show **A** c-Fos expression in the hippocampus and **B** MAP2 expression in the cortex. The scale bar represents 100 µm. **C** Quantification of staining intensity. Bar graphs represent means ± SEM (*n* = 5 mice per group). Statistical significance was determined using one-way ANOVA followed by Tukey’s post hoc test. *p < 0.05, **p < 0.01, and *p < 0.001. Genotypes: WT (wild-type), Syn (α-Syn transgenic), PME (PME-1 overexpressing), PME-Syn (PME-1 and α-Syn Triple transgenic), LCMT (LCMT-1 overexpressing), and LCMT-Syn (LCMT-1 and α-Syn Triple transgenic).

To evaluate the effects of PP2A methylation on neuronal function and integrity in the context of α-Syn pathology, we analyzed hippocampal neuronal activity and cortical neuronal integrity in brain sections from each group. Quantification of c-Fos immunoreactivity in the hippocampus showed that both α-Syn and PME-1/α-Syn mice had significantly reduced c-Fos expression compared to WT mice, with the PME-1/α-Syn

group showing more pronounced reduction (Fig. 2A, C). In contrast, LCMT-1/α-Syn mice displayed c-Fos levels equivalent to those in WT mice, indicating preservation of neuronal function (Fig. 2A, C). A similar profile was seen with MAP2 immunostaining of the cortex. While α-Syn transgenic mice exhibit significant loss of dendritic integrity relative to WT mice, PME-1/α-Syn mice demonstrated even greater MAP2 depletion and neuronal fragmentation. Conversely, LCMT-1/α-Syn mice showed significant preservation of MAP2 expression, suggesting a neuroprotective effect of enhanced PP2A methylation (Fig. 2B, C). Taken together, these results indicate that PME-1 overexpression exacerbates α-Syn–induced neuronal dysfunction and structural damage, whereas LCMT-1 overexpression preserves both neuronal activity and morphology in the context of α-Syn pathology.

### PP2A methylation modulates the neuroinflammatory response in α-Syn transgenic mice

**Fig. 3:**
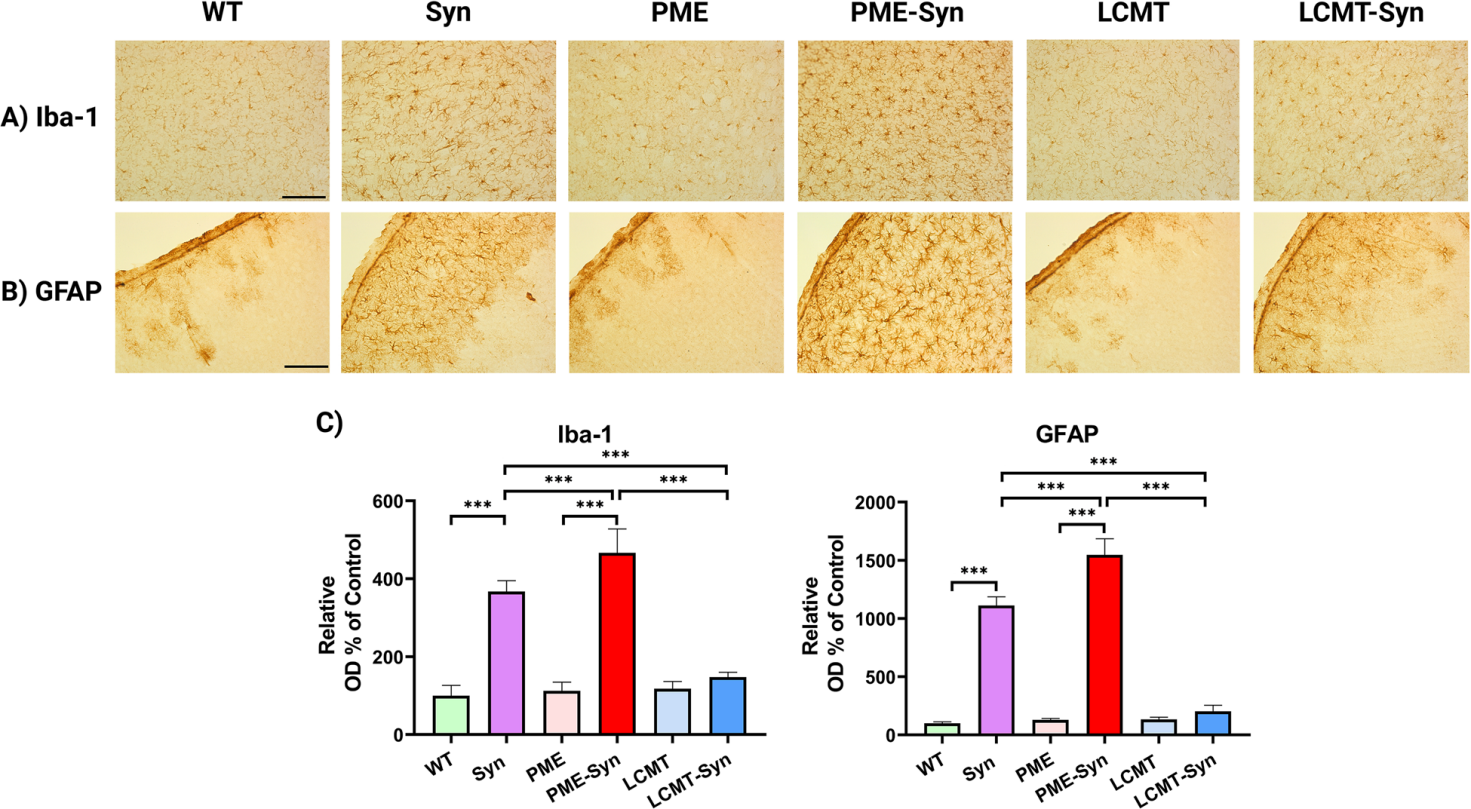
PP2A methylation attenuates the neuroinflammatory response to α-Syn. Representative IHC images of cortical sections showing neuroinflammatory markers, **A** Iba-1, and **B** GFAP. The scale bar represents 100 µm. **C** Quantification of staining intensity. Bar graphs display means ± SEM (*n* = 5 mice per group). Statistical significance was assessed using one-way ANOVA followed by Tukey’s post hoc test. ***p < 0.001. Genotypes: WT (wild-type), Syn (α-Syn transgenic), PME (PME-1 overexpressing), PME-Syn (PME-1 and α-Syn Triple transgenic), LCMT (LCMT-1 overexpressing), and LCMT-Syn (LCMT-1 and α-Syn Triple transgenic).

Given the well-established link between α-Syn pathology and neuroinflammation in PD [24] and animal models of synucleinopathies [25], the neuroinflammatory response was evaluated by analyzing the levels of Iba-1, a marker of microglial activation, and GFAP, a marker of astrocytic reactivity. Iba-1 and GFAP immunoreactivity were significantly increased in the cortex of α-Syn (Tg) mice, indicating elevated glial activation in response to α-Syn pathology. This effect was exacerbated in PME-1/α-Syn mice, which displayed a further increase in Iba-1 and GFAP expression. In contrast, LCMT-1 overexpression attenuated microgliosis and astrogliosis, as evidenced by a reduction in Iba-1 and GFAP immunoreactivity compared to both α-Syn (Tg) and PME-1/α-Syn mice (Fig. 3). These findings suggest that PP2A methylation plays a crucial role in modulating neuroinflammatory responses to α-Syn overexpression, with PME-1 exacerbating and LCMT-1 mitigating these pathological processes.

### PP2A methylation improves the behavioral phenotype of α-Syn transgenic mice

**Fig. 4:**
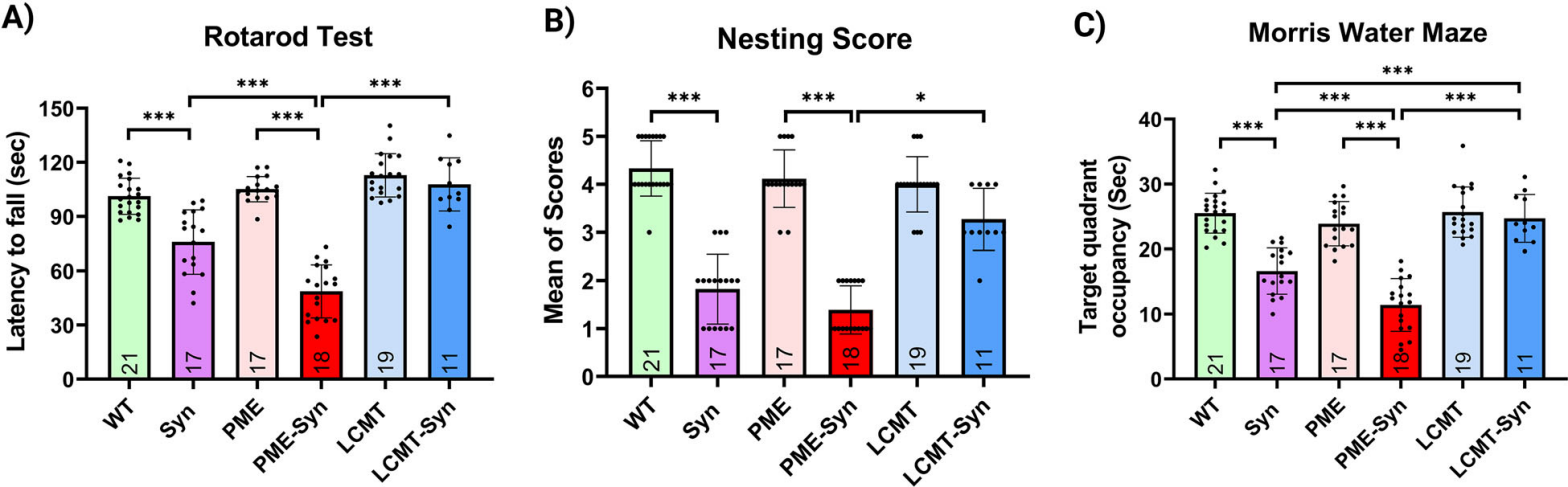
Mice co-expressing PME-1 and α-Syn in the forebrain exhibit greater behavioral deficits across multiple tests. **A** Rotarod, **B** nesting behavior, and **C** Morris water maze assessments. Bar graphs represent means ± SEM. Statistical significance was analyzed using one-way ANOVA followed by Tukey’s post hoc test for parametric data or the Kruskal-Wallis test for non-parametric data. *p < 0.05 and ***p < 0.001. Genotypes: WT (wild-type), Syn (α-Syn transgenic), PME (PME-1 overexpressing), PME-Syn (PME-1 and α-Syn Triple transgenic), LCMT (LCMT-1 overexpressing), and LCMT-Syn (LCMT-1 and α-Syn Triple transgenic). Sample sizes (*n*) are indicated within the bars.

As expected, α-Syn transgenic mice exhibited motor and cognitive impairments, performing worse than control mice (CamK2a, PME-1, or LCMT-1 single-transgenic mice). Our findings revealed that triple-transgenic mice expressing PME-1 and α-Syn (PME-1/α-Syn) exhibited exacerbated α-Syn-induced disease phenotype, as evidenced by significantly impaired motor and cognitive performance on the rotarod and Morris water maze compared to α-Syn single transgenic mice (Fig. 4). In contrast, α-Syn transgenic mice overexpressing LCMT-1 (LCMT-1/α-Syn) demonstrated improved behavior, particularly in the Morris Water Maze, performing better than α-Syn transgenic and PME-1/α-Syn mice, and comparably to WT mice, consistent with a protective effect of PP2A methylation (Fig. 4). Analysis of swim speed during hidden platform training followed a pattern similar to that seen during probe trial performance (Supplementary Fig. 1), suggesting that the differences observed in the Morris water maze performance may have been influenced, at least in part, by motor function. Both male and female mice were included in these behavioral assessments, and after confirming the lack of sex-based differences (Supplementary Fig. 2A), their data were combined for further analysis.

### PP2A methylation prevents pS129-α-Syn accumulation and propagation in the PFF model of synucleinopathy

**Fig. 5:**
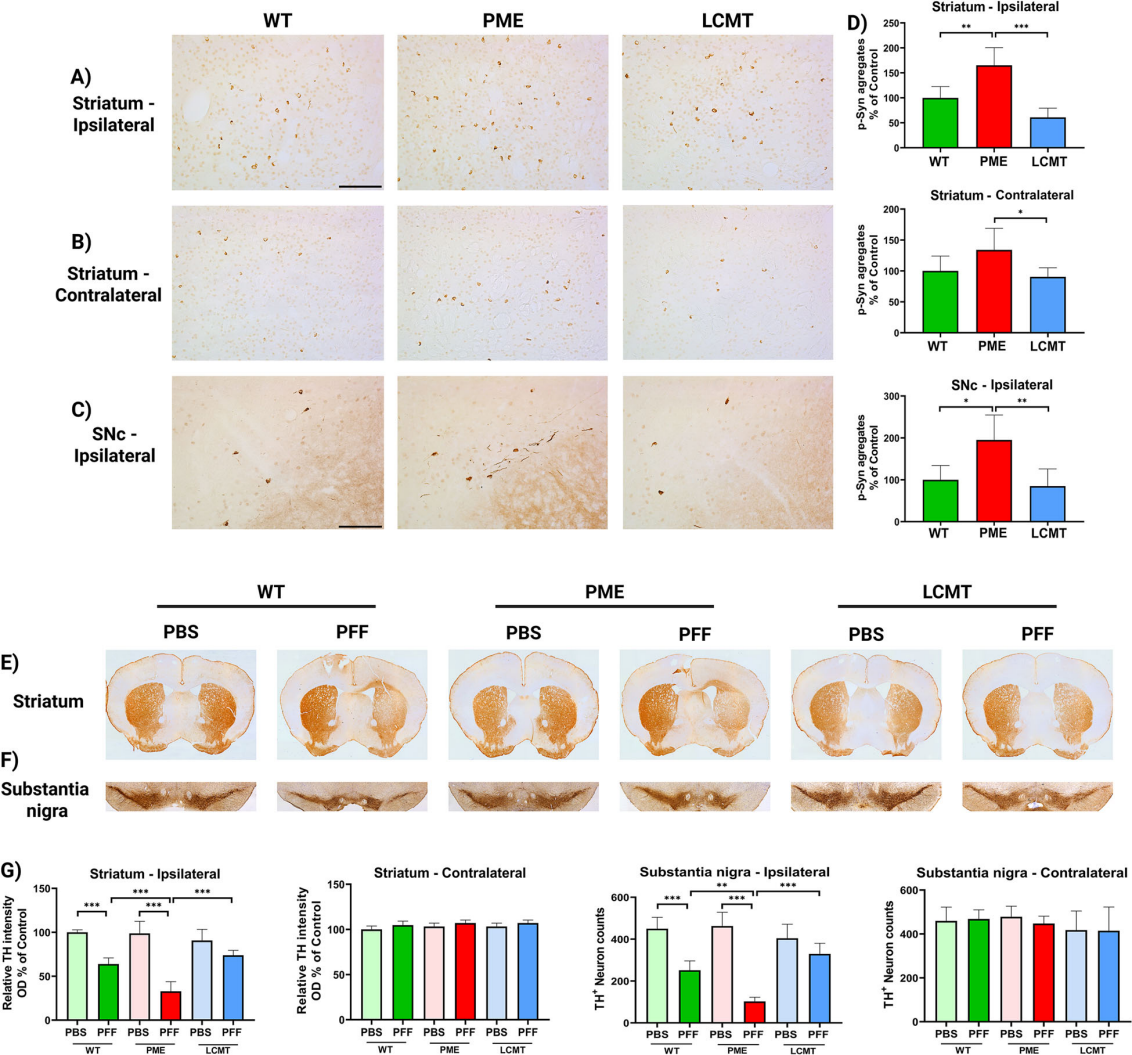
PP2A methylation prevents the formation of p-α-Syn aggregates and protects dopaminergic neurons from degeneration in PFF-induced α-Syn seeding. Representative immunohistochemical images showing **A** p-α-Syn staining in the ipsilateral striatum, **B** contralateral striatum, and **C** ipsilateral substantia nigra. Scale bars: 100 µm. **D** Quantification of p-α-Syn aggregates. Representative immunohistochemical images of TH staining in **E** striatum and **F** substantia nigra. **G** Quantification of TH-positive neurons and terminals in the regions of interest. Bar graphs represent means ± SEM (*n* = 5 mice per group). Statistical significance was determined using one-way ANOVA followed by Tukey’s post hoc test. *p < 0.05; **p < 0.01; ***p < 0.001. Genotypes: WT (wild-type), PME (PME-1 overexpressing), and LCMT (LCMT-1 overexpressing).

To further investigate the effect of PP2A methylation on α-Syn–mediated pathology, particularly the propagation of its aggregated form, we employed the unilateral PFF model of synucleinopathy. In this model, injection of PFF into the striatum functions as a seed to initiate the aggregation of endogenous α-Syn, thereby recapitulating the progressive spread of pathology characteristic of synucleinopathies [26]. Immunohistochemical staining for pS129-α-Syn revealed significant accumulation of p-α-Syn aggregates in both the ipsilateral and contralateral striata, as well as the ipsilateral substantia nigra, of PFF-injected mice, confirming the pathological impact of the PFF model (Fig. 5A–D). No p-α-Syn aggregates were observed in the brains of PBS-injected mice (Supplementary Fig. 3). PME-1 mice exhibited significantly more pS129-α-Syn aggregates in the ipsilateral striatum following PFF injection compared with WT mice, whereas LCMT-1 mice displayed markedly less pS129-α-Syn accumulation relative to PME-1 expressing mice. Additionally, in the contralateral striatum, pS129-α-Syn accumulation was significantly greater in PME-1 mice compared with LCMT-1 mice. PME-1 transgenic mice also had greater accumulation of pS129-α-Syn aggregates in the ipsilateral substantia nigra following PFF injection compared with WT mice, while the LCMT-1 group exhibited a marked reduction in aggregates compared to PME-1 mice (Fig. 5A–D). These findings support a protective role of PP2A methylation against α-Syn aggregation and propagation in the PFF model.

### PME-1 overexpression exacerbates PFF-induced nigrostriatal dopaminergic neurodegeneration, while LCMT-1 overexpression mitigates it

To evaluate the impact of PP2A methylation on nigrostriatal integrity, we performed tyrosine hydroxylase immunostaining to visualize dopaminergic terminals in the striatum and dopaminergic neurons in the substantia nigra. PFF-injected WT and PME-1 mice exhibited a significant reduction in TH signal intensity in the ipsilateral striatum and a decreased number of TH-positive neurons in the ipsilateral substantia nigra compared to PBS-injected controls (Fig. 5E–G). Further analysis revealed that PME-1 overexpression heightened the susceptibility to PFF-induced dopaminergic degeneration. Specifically, PME-1 mice injected with PFF exhibited a more pronounced loss of dopaminergic terminals in the ipsilateral striatum compared to WT and LCMT-1 groups, indicating increased vulnerability associated with PP2A demethylation (Fig. 5E, G). Similarly, automated quantification of TH-positive neurons in the substantia nigra pars compacta (SNc) revealed a significant reduction in PME-1 mice compared with both WT and LCMT-1 groups following PFF injection (Fig. 5F, G), confirming their increased sensitivity to α-Syn pathology. On the other hand, LCMT-1 mice were less susceptible to PFF injections compared to PME-1 mice, and this was evident in the ipsilateral striatum and nigra where no significant differences were detected between PBS and PFF-injected LCMT-1 mice (Fig. 5E–G). These findings demonstrate that PP2A demethylation in PME-1 mice exacerbates PFF-induced dopaminergic neurodegeneration, while PP2A methylation in LCMT-1 mice protects these neurons, thus underscoring the role of impaired PP2A methylation in α-Syn–driven nigrostriatal damage.

### PP2A methylation attenuates neuroinflammatory responses to PFF injection

**Fig. 6:**
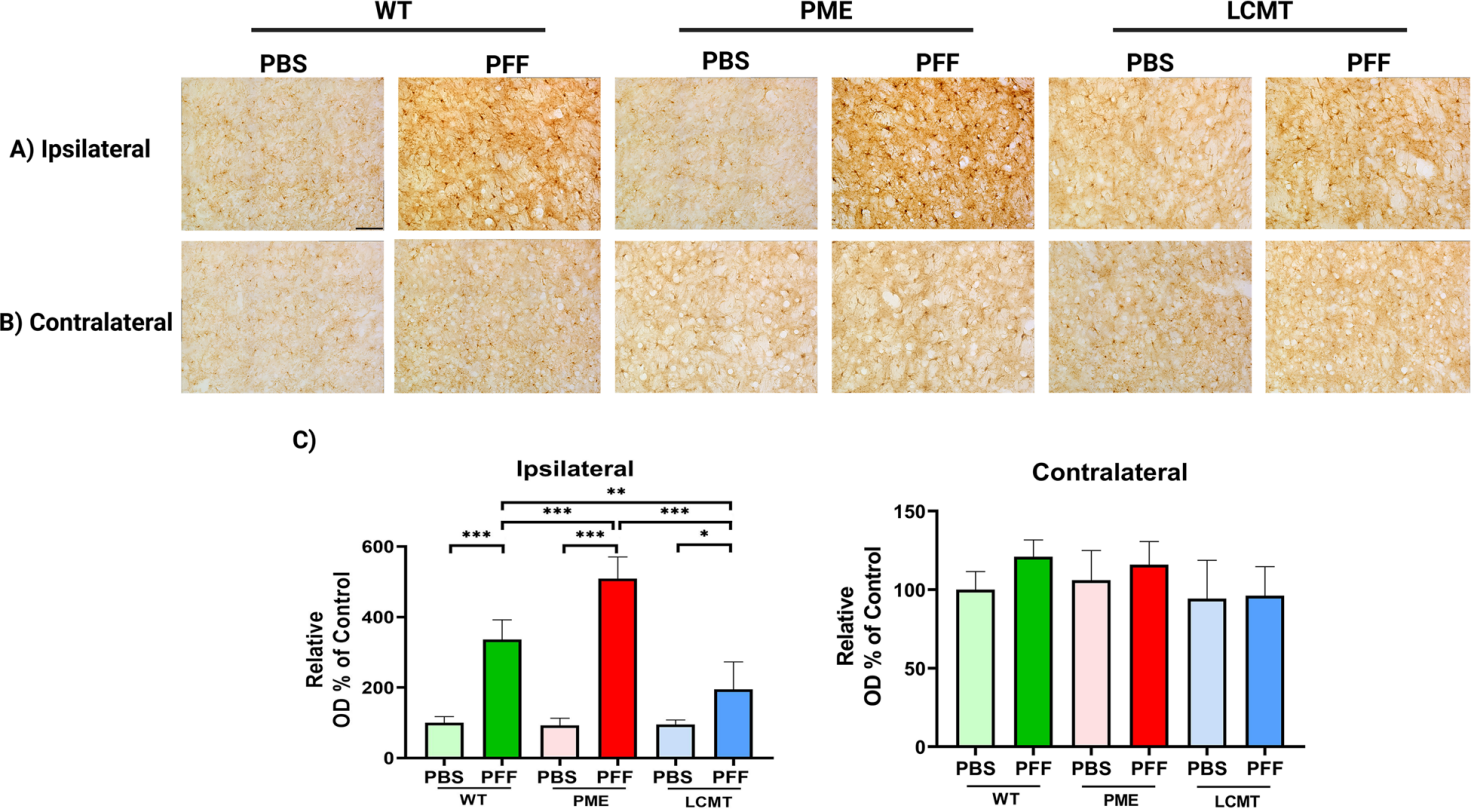
α-Syn PFF-induced neuroinflammation is reduced by promoting PP2A methylation. Representative immunohistochemistry images showing Iba-1 staining in the **A** ipsilateral, **B** contralateral striatum. Scale bar: 100 µm. **C** Quantification of Iba-1 optical density. Bar graphs represent means ± SEM (*n* = 5 mice per group). Statistical significance was determined using one-way ANOVA followed by Tukey’s post hoc test. *p < 0.05; **p < 0.01; ***p < 0.001. Genotypes: WT (wild-type), PME (PME-1 overexpressing), and LCMT (LCMT-1 overexpressing).

Given the observed differences in pS129-α-Syn aggregate accumulation and neurodegeneration across experimental groups, we next examined neuroinflammation, a process closely linked to these pathological features [27]. Immunohistochemical staining for Iba-1, a widely used marker of microglial activation, revealed a significant increase in Iba-1–positive cells in the ipsilateral striatum of all three mouse lines (WT, PME-1 and LCMT-1) following PFF injection compared to PBS-injected controls (Fig. 6A, C). In contrast, no significant microglial activation was detected in the contralateral striata of PFF-injected mice (Fig. 6B, C) or in either hemisphere of PBS-treated mice (Fig. 6A, C), confirming that the inflammatory response was specifically triggered by PFF-induced pathology.

Notably, PME-1 over-expressing mice exhibited a significantly higher density of Iba-1–positive microglia in the ipsilateral striatum relative to both WT and LCMT-1 over-expressing mice post-PFF injections, indicating that PP2A demethylation enhances microglial reactivity to pathological α-Syn. Conversely, LCMT-1 over-expressing mice showed markedly fewer Iba-1–positive microglia compared to both WT and PME-1 mice, suggesting that enhanced PP2A methylation attenuates the neuroinflammatory response to PFF (Fig. 6A, C). These findings underscore the regulatory role of PP2A methylation in modulating microglial activation in response to α-Syn pathology.

### PP2A methylation improved the behavioral phenotype in the PFF model of synucleinopathy

**Fig. 7:**
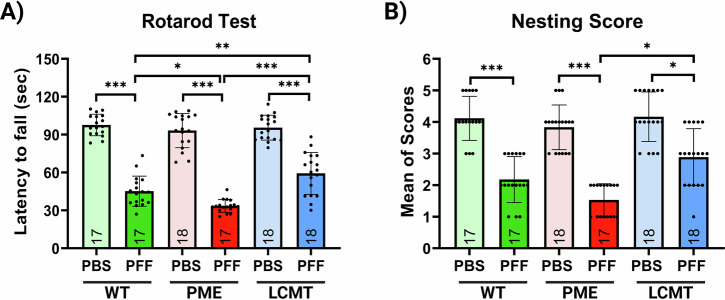
PP2A activation prevents PFF-induced behavioral phenotype. **A** Rotarod and **B** nesting behavior tests. Bar graphs represent means ± SEM. Statistical significance was analyzed using one-way ANOVA followed by Tukey’s post hoc test for parametric data or the Kruskal-Wallis test for non-parametric data. *p < 0.05, **p < 0.01, and ***p < 0.001. Genotypes: WT (wild-type), PME (PME-1 overexpressing), and LCMT (LCMT-1 overexpressing). Sample sizes (*n*) are indicated within the bars.

To assess the impact of PP2A methylation on behavioral phenotype in the PFF model, motor function was assessed using the Rotarod test, which measures balance and coordination based on the time animals remain on the rotating rod. Across all genotypes, PFF-injected mice exhibited a significant decline in motor performance compared to PBS-injected controls. Notably, PME-1 over-expressing mice showed a further exacerbation of motor deficits following PFF injection relative to PFF-injected WT and LCMT-1 mice, suggesting that impaired PP2A methylation worsens motor dysfunction. In contrast, LCMT-1 over-expressing mice demonstrated notable resistance to PFF-induced motor impairment, performing significantly better than both PFF-injected WT and PME-1 mice (Fig. 7A).

Consistent with these findings, nesting behavior was significantly impaired in PFF-injected mice across all groups compared with their PBS-injected littermates. This deficit was most pronounced in PME-1 overexpressing mice, which exhibited a further reduction in nesting performance following PFF treatment. Conversely, LCMT-1 over-expressing mice maintained significantly better nest-building activity despite PFF injection, outperforming PME-1 mice (Fig. 7B). As with the transgenic models described above, the PFF model studies included both male and female mice, and because no sex differences were found (Supplementary Fig. 2B), their data were combined and analyzed together. Together, these results support a protective role for PP2A methylation in mitigating PFF-induced behavioral deficits and disease progression.

## Discussion

The present study underscores the critical role of PP2A methylation in regulating α-Syn pathology, neurodegeneration and neuroinflammation across two synucleinopathy models. In Triple transgenic mice expressing α-Syn, PP2A demethylation via PME-1 overexpression led to elevated pS129-α-Syn aggregation, neuronal damage, and robust neuroinflammatory responses. Conversely, overexpression of LCMT-1, which promotes PP2A methylation, significantly reduced these α-Syn–associated pathological features by nine months of age. These molecular and cellular changes were reflected in the behavioral phenotype, with PME-1 mice displaying greater impairments while LCMT-1 mice exhibited preserved performance. Similarly, in the α-Syn PFF model six months post-injection, PME-1 overexpressing mice had pronounced α-Syn aggregation, neurodegeneration and neuroinflammation. In contrast, LCMT-1 overexpression was protective and counteracted α-Syn-mediated pathological changes. Again, these molecular alterations were mirrored in behavioral outcomes.

Indices of PP2A activity are significantly diminished in postmortem brains of individuals with multiple neurodegenerative diseases. In PD and DLB, pronounced reduction in methylated PP2A levels has been observed in the substantia nigra and frontal cortex, accompanied by a significant decrease in the ratio of methylated to demethylated PP2A. This imbalance is linked to reduced expression of the methyltransferase LCMT-1 and elevated levels of the methyeserase PME-1 [20]. Similar impairments in PP2A have been reported in Alzheimer’s disease, where both its expression and enzymatic activity are reduced in postmortem brain tissue [28–31]. Consistent with this observation, experimental manipulation of PP2A methylation in mice through overexpression of LCMT-1 or PME-1 modulates susceptibility to oligomeric tau–induced cognitive and electrophysiological impairments in opposite directions, supporting a functional link between PP2A methylation status and tau toxicity[32]. Multiple pathogenic mechanisms impair the function of PP2A in neurodegenerative diseases, particularly in synucleinopathies. Among these, oxidative stress plays a central role, with elevated oxidative markers observed in both PD [33] and DLB brains [34]. PP2A is highly sensitive to redox imbalance [35], largely due to the oxidation of two vicinal cysteine residues near its catalytic site, which can form disulfide bonds and disrupt both the structural integrity of the enzyme and its interaction with LCMT-1 [36]. Even low micromolar concentrations of hydrogen peroxide can block PP2A methylation by LCMT-1, thereby diminishing its activity [37]. Notably, overexpression of α-Syn in a cellular model, and particularly its pathogenic A53T mutant, elevates reactive oxygen species levels [38], which in turn exacerbates oxidative inhibition of PP2A [35, 37]. In a rat model, viral overexpression of α-Syn in the striatum reduces the expression of PP2A B and C subunits and overall enzymatic activity, associated with enhanced α-Syn phosphorylation, aggregation, neuroinflammation, and neuronal loss [39]. In addition, PP2A activity is impacted directly by aggregated α-Syn, as demonstrated in vitro with recombinant α-Syn protein [19]. Another contributing factor to PP2A dysregulation is deficiency of glucocerebrosidase (GCase), a lysosomal enzyme genetically associated with PD and found to be reduced in sporadic cases [40, 41]. Loss of GCase activity promotes accumulation of phosphorylated and oligomeric α-Syn, while also reducing PP2A methylation and activity, particularly in brain regions vulnerable to synucleinopathy, as shown in both aged non-human primates and neuronal cell models [11, 42]. Together, these findings reveal that oxidative stress, α-Syn aggregation, and lysosomal dysfunction converge on a common pathway of impaired PP2A regulation, contributing to the molecular pathogenesis of PD and DLB.

Given the pathological mechanisms outlined above and the central role of PP2A methylation in modulating α-Syn phosphorylation, enhancing PP2A activity has emerged as a promising therapeutic strategy in neurodegeneration. Pharmacological interventions targeting PP2A methylation have shown encouraging results. Eicosanoyl-5-hydroxytryptamide (EHT), a naturally occurring serotonin derivative found in coffee, inhibits PME-1, thereby preserving PP2A methylation and stabilizing the active PP2A holoenzyme. In α-Syn transgenic mice, chronic administration of EHT significantly reduced pS129-α-Syn levels, attenuated α-Syn aggregation, preserved neuronal integrity, dampened neuroinflammatory responses, and improved motor function [25]. Beyond synucleinopathies, EHT also preserves PP2A activity in AD models, reducing tau hyperphosphorylation and intraneuronal Aβ accumulation, while rescuing tau pathology and minimizing cognitive deficits [43]. It further protects against Aβ-induced impairments in long-term potentiation via the same mechanism [44]. Additional PP2A activating agents have shown promise in rodent models of AD [45–48].

Together, these findings provide strong pharmacological support for the mechanistic framework established in the present study and underscore the therapeutic relevance of targeting the PP2A signaling pathway. While our data highlight the importance of PP2A methylation in modulating α-Syn pathology, the current study does not entirely exclude potential off-target effects or the long-term safety of enhancing PP2A activity, although the administration of the PME-1 inhibitor EHT to mice for 9 months is well tolerated [9, 25]. In conclusion, the results of this study provide direct in vivo validation of PP2A methylation as a key modulator of α-Syn-driven pathology. Collectively, our data demonstrate that PP2A methylation is a critical regulatory mechanism in α-Syn pathology, neuroinflammation, and neurodegeneration. Specifically, PME-1 overexpression exacerbates α-Syn aggregation, neuronal loss, and inflammatory responses, while LCMT-1 overexpression mitigates these pathological outcomes. These findings position PP2A as a key upstream regulator of α-Syn phosphorylation and support the notion that enhancing PP2A phosphatase activity may offer disease-modifying potential in PD and related disorders. Accordingly, future efforts to develop selective PP2A modulators will benefit from optimizing therapeutic efficacy while assessing and avoiding any off-target effects.

## Materials and methods

### Animals

Transgenic mice overexpressing PME-1 or LCMT-1 transgene were generated under the control of a synthetic tetO promoter, which is active only in the presence of a second transgene encoding the synthetic tetracycline-responsive transactivator (tTA), driven by a fragment of the calcium/calmodulin-dependent protein kinase II alpha (CaMK2a) gene promoter expressed in forebrains neuron [23]. Based on the initial study that generated and characterized these mouse models, PME-1 overexpression reduces PP2A methylation, whereas LCMT-1 overexpression increases PP2A methylation and alters PP2A holoenzyme assembly and functional activity[23].

These mice were maintained by breeding male transgenic mice with female C57BL/6 mice (The Jackson Laboratory #000664). Additionally, Syn (Tg), which overexpresses human wild-type α-Synuclein under the Thy1 promoter, was used in this study. These mice were bred on a BDF1 background by mating heterozygous transgenic females with wild-type (WT) BDF1 males. The BDF1 strain is a hybrid generated by crossing female C57BL/6 mice (The Jackson Laboratory # 100006) with male DBA/2 mice (Charles River Laboratories #026). Female Syn (Tg) mice were bred with either PME-1/CaMK2a or LCMT-1/CaMK2a double transgenic male mice, resulting in offspring with various genotypes, including triple transgenic (Syn/PME-1/CaMK2a or Syn/LCMT-1/CaMK2a), double transgenic (PME-1/CaMK2a or LCMT-1/CaMK2a), and single transgenic (Syn or PME-1 or LCMT-1 or CaMK2a) mice. Both male and female mice from these genotypes were included in the study.

In addition to these triple transgenic mice, we employed another model of synucleinopathy, namely the α-Syn preformed fibrils propagation model. For this, male double-transgenic mice, either PME-1/CaMK2a or LCMT-1/CaMK2a mice, were crossed with female wild-type C57BL/6 mice to generate PME-1/CaMK2a, LCMT-1/CaMK2a, and C57BL/6 offspring. Both male and female double-transgenic mice were used in this model. At two months of age, mice were unilaterally inoculated with α-Syn PFF in the right striatum to induce pathological α-Syn aggregation and propagation. Wild-type littermates were used as controls in all evaluations. All animals were housed and maintained in accordance with the guidelines of the National Institutes of Health and with the approval of Rutgers – Robert Wood Johnson Medical School Institutional Animal Care and Use Committee (IACUC).

In both models, animals were randomly assigned to experimental groups after genotyping, and investigators were blinded to group allocation to the extent possible during data collection and analysis. Sample sizes for behavioral studies and brain analyses were determined based on our prior experience with these models and on data from our previously published studies and those of others [25, 49, 50].

### Reagents

The primary antibodies used are as follows: anti-pS129-α-syn (#015–25191, FUJIFILM WAKO, 1:5000), anti-Iba-1 (#019–19741, FUJIFILM WAKO 1:1000), anti-tyrosine hydroxylase (TH) (T-2928, Sigma, 1:1500 in striatum, and 1:2000 in the substantia nigra), anti-microtubule associated protein 2 (MAP2) (ab32454, Abcam), anti-c- fos (sc-52, Santa Cruz, 1:100), anti-glial fibrillary acidic protein (GFAP) (GA524, Dako). Secondary antibodies used are as follows: Goat anti-rabbit IgG (H + L) secondary antibody-Biotin (65-6140, Invitrogen), Goat anti-mouse IgG secondary antibody-Biotin (B7264, Sigma-Aldrich), Alexa Fluor™ 488 (A11008, Invitrogen). Hoechst (Tocris Bioscience, MO, USA), Vectastain Elite ABC kit (PK-6100, Vector Laboratories), and 3,3’-diaminobenzidine (DAB) (D4293, Sigma-Aldrich).

### α-Syn PFF preparation

Mouse α-Syn cDNA, encoded by the pT7-7 plasmid, was used for the transformation of *Escherichia coli* BL21(DE3) (Invitrogen Inc.). Transformed *E. coli* was cultured in LB medium and incubated at 37 °C. When the optical density at 600 nm (OD600) reached 0.8, α-Syn expression was induced by adding 1 mL of 1 M isopropyl β-D-1-thiogalactopyranoside (IPTG). Following a 4-hour incubation, bacterial cells were harvested by centrifugation, and the pellet was resuspended in 25 mL of phosphate-buffered saline (PBS). Cells were homogenized using a Branson-150 sonicator, and the lysate was subjected to centrifugation at 24,000 × *g* for 30 min. The resulting supernatant was collected, and streptomycin sulfate was added to a final concentration of 10 mg/mL, followed by incubation at 4 °C for 30 min with continuous stirring. After a second centrifugation step, ammonium sulfate was added to the supernatant at a concentration of 0.361 g/mL, and the mixture was incubated at 4 °C for 60 min under stirring. The resulting pellet was collected by centrifugation and resuspended in 15 mL PBS, followed by heat treatment in a boiling water bath for 15 min. After cooling, the solution underwent a final centrifugation at 24,000 × *g* for 30 min, and the supernatant was collected. The α-Synuclein-containing solution was dialyzed against 25 mM Tris buffer (pH 7.7) and subsequently purified using fast protein liquid chromatography (FPLC, GE Healthcare). The purified α-Syn was lyophilized and later reconstituted in PBS at a final concentration of 5 mg/mL. To induce fibrillation, the solution was incubated at 37 °C under constant agitation at 1000 rpm for 1 week using a thermomixer. The formation of fibrillar α-Syn was confirmed using the thioflavin-T assay [25, 49].

### Stereotaxic inoculation of α-Syn PFF

The α-Syn PFF solution was diluted in PBS and sonicated at 30% amplitude for 30 cycles (0.5 s on, 0.5 s off) [26, 49]. Eight-week-old mice were anesthetized with an intraperitoneal injection of ketamine/xylazine (90/4.5 mg/kg). The animals were then secured on a stereotaxic apparatus (Stoelting, Wood Dale, IL). A sagittal incision was made along the scalp, and a small hole was drilled into the skull at the predetermined coordinates. Injections were performed using a Hamilton syringe connected to a Quintessential Stereotaxic Injector (Stoelting, Wood Dale, IL). A total volume of 2.5 µL containing 5 µg of α-Syn PFF or PBS was injected into the right striatum at stereotaxic coordinates ( + 0.2 mm relative to Bregma, +2.0 mm lateral from the midline, +2.6 mm beneath the dura) [26]. To minimize reflux, the injection needle was kept in place for 5 min following each infusion before being withdrawn. Following the procedure, mice were allowed to recover on a 37 °C heating pad and were closely monitored.

### Immunohistochemistry and immunofluorescence

Immunohistochemical procedures were conducted following previously established protocols [25]. Mice were perfused transcardially with PBS, after which brains were extracted and post-fixed in 10% formalin (Sigma, St. Louis, MO) at 4 °C overnight. The fixed brains were cryoprotected in 30% sucrose until they sank, then sectioned coronally at a thickness of 30–35 µm using a Leica CM3050 S cryostat. Serial sections were collected at regular intervals for further immunohistochemical analysis.

Free-floating brain sections were selected for immunohistochemical analysis. For antigen retrieval, sections were incubated in sodium citrate at 80 °C for 20 min. To quench endogenous peroxidase activity, sections were treated with 0.3% hydrogen peroxide (H₂O₂, Sigma-Aldrich) for 30 min. Blocking was performed using 5% normal serum and 0.3% Triton X-100 in PBS. Sections were then incubated overnight at 4 °C with the primary antibody, followed by a one-hour incubation at room temperature with a biotinylated secondary antibody. Signal amplification was achieved using the Vectastain Elite ABC kit (Vector Laboratories, Burlingame, CA), and color development was performed using 3,3’-diaminobenzidine (DAB, Sigma-Aldrich). Fluorescent secondary antibodies were applied for an additional one-hour incubation at room temperature when necessary. Images were acquired using a Leica DMi8 microscope. The intensity of immunostaining and the number of positive cells were analyzed and quantified using ImageJ software.

### Image J analysis

Phosphorylated α-Syn (p-α-Syn) aggregates were quantified in the cortex, striatum, and substantia nigra pars compacta (SNc), with four corresponding regions analyzed in each of the five sections per animal. For p-α-Syn and c-Fos staining in the hippocampus, regions of interest (ROIs) of consistent size covering CA2 and CA3 were uniformly applied across all images, and optical density (OD) was measured for both markers. GFAP and MAP2 staining were assessed in four matching regions per section across five sections per animal. Similarly, Iba-1 and TH staining in the striatum were analyzed in four corresponding regions per section. All staining experiments were conducted on five animals per group.

### Automated TH-positive neuron count

Quantification of TH-positive neurons in the SNc was performed using an unbiased, automated artificial intelligence platform (Aiforia®, Fimmic Oy, Helsinki, Finland) on three to four sections per brain. High-resolution images (0.3 μm per pixel) were uploaded to the Aiforia cloud platform, where a specifically trained algorithm identified TH-positive cells in the substantia nigra pars compacta. This computer-assisted approach, leveraging supervised machine learning and automated image recognition, has been extensively described and validated [25, 49, 51–53]. For all the staining’s 5 brains per group are analyzed.

### Behavioral tests

Behavioral tests on triple Tg mice were performed at nine months of age, a time point commonly used to capture the onset of robust pathological and behavioral deficits in α-synuclein transgenic models [22, 54]. In the PFF model, behavioral assessments were conducted six months post-injection, corresponding to the established emergence of α-synuclein pathology and motor impairment in this model [25, 26].

#### Rotarod test

The rotarod test was performed using an automated system (Med Associates Inc. or TSE System). Mice underwent training three times per day over four consecutive days following the standard laboratory protocol. Each training session lasted up to 180 s, with the rod accelerating from 2 to 40 rpm during the acquisition phase and from 4 to 40 rpm during probe trials. On the 5th day, mice underwent three probe trials under the same conditions, and the average latency to fall was recorded as a measure of motor performance.

#### Nesting behavior test

Nesting behavior is an indicator of well-being and sensorimotor function. Each mouse was individually housed in a cage containing a 5 cm tightly packed cotton square Nestlet (Ancare). After 16 h, nest quality was assessed using a blinded scoring system, ranging from 1 (untouched Nestlet) to 5 (fully shredded Nestlet), based on established criteria [55].

#### Morris water maze

Spatial learning and memory were assessed using the Morris Water Maze test. The test was conducted in a tank filled with warm water, with spatial cues placed around the tank to facilitate navigation. The tank was conceptually divided into quadrants, and a hidden platform was positioned within one of them. A ceiling-mounted video camera connected to a video-tracking system recorded the animals’ movements, enabling precise analysis of their interactions with the platform.

Before the main trials, animals underwent a pre-acquisition training phase. On the first day, a visible platform was introduced to allow the mice to familiarize themselves with the task and platform location. Following this, the platform was submerged but kept in a fixed position for three consecutive training days. Each day, mice completed four trials, with release points varying across quadrants to encourage reliance on spatial cues and memory. On the final day, a probe test was conducted in which the platform was removed from the tank to evaluate spatial memory retention. The time each mouse spent in the previously learned target quadrant was recorded as an indicator of memory consolidation and spatial learning ability.

### Statistical analysis

Bar graphs display the data as means ± standard error of the mean (SEM). Statistical comparisons between group means were conducted using one-way analysis of variance (ANOVA) followed by Tukey’s post hoc test, or the Kruskal-Wallis test for non-parametric data when applicable. Assumptions of variance and data distribution were evaluated using GraphPad Prism prior to statistical testing, and appropriate parametric or non-parametric tests were applied accordingly. A *p*-value below 0.05 was considered statistically significant.

## Supplementary information

Supplementary Information

## Data Availability

The datasets used and/or analyzed during the current study are available from the corresponding author upon reasonable request.
