# Supplementary material for: Protein phosphatase 2A methylation state impacts α-synucleinopathy in mouse models
Source: Cell Death Discov. 2026 Mar 24;12:172. doi: 10.1038/s41420-026-03045-7 (PMC13039847; doi:10.1038/s41420-026-03045-7)
Supplement: Supplementary file 1 — Supplementary Information [file 41420_2026_3045_MOESM1_ESM.pdf]

Supplementary Information

Protein Phosphatase 2A Methylation State Impacts α-Synucleinopathy in Mouse Models

Santhosh Maddila,[1]* Kambiz Hassanzadeh,[1]* Jun Liu,[1] Jie Zhang,[1] Russell E. Nichols,[2] M. Maral Mouradian[1]

*These authors contributed equally to the study

[1] Robert Wood Johnson Medical School Institute for Neurological Therapeutics, and Department of Neurology, Rutgers Biomedical and Health Sciences, Piscataway, NJ 08854, USA.

[2] Department of Pathology and Cell Biology, and The Taub Institute for Research on Alzheimer's Disease and the Aging Brain, Columbia University, New York, NY, 10032, USA

Corresponding author

M. Maral Mouradian, RWJMS Institute for Neurological Therapeutics, 683 Hoes Lane West, Room 180, Piscataway, NJ 08854, USA. E-mail: m.mouradian@rutgers.edu; Phone: 732-235-4772; Fax: 732-235-4773

Fig. S1

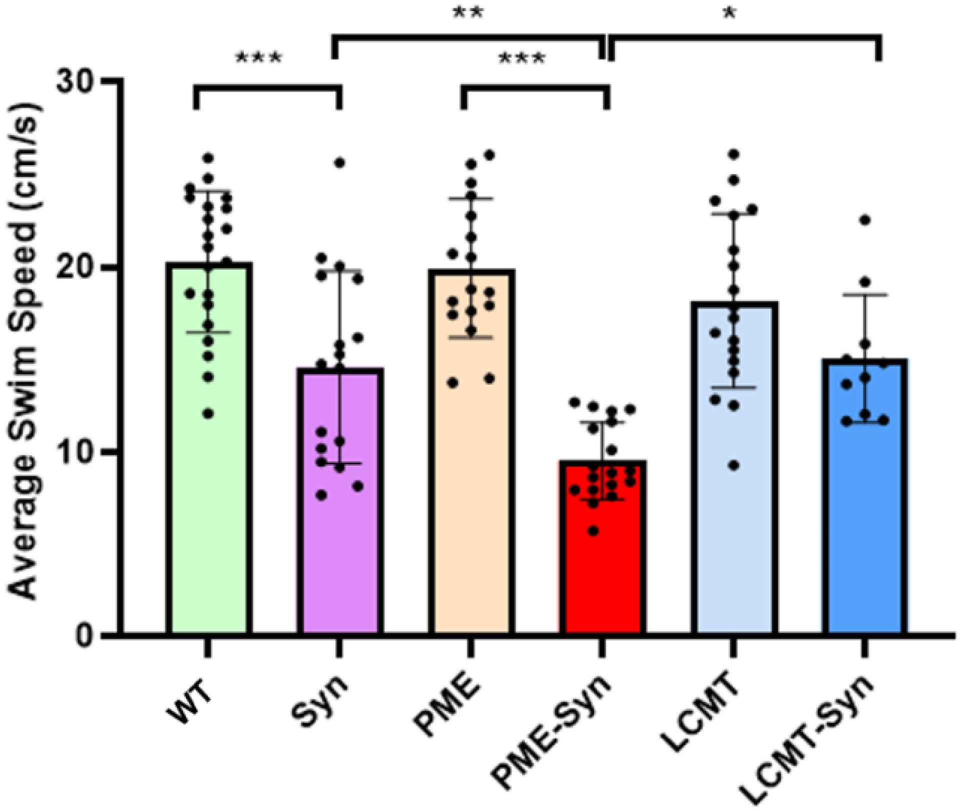

Figure S1. Average swim speed across experimental groups in the Morris Water Maze test. α-Syn transgenic mice showed significantly reduced swim speed compared to wild-type mice. PME-Syn mice swam significantly slower than α-Syn mice as well as LCMT-Syn mice. No significant differences were observed between LCMT and LCMT-Syn groups. Bar graphs show means ± SEM with individual data points overlaid. Statistical analysis was performed using one-way ANOVA followed by Tukey's post hoc test. Genotypes: WT (wild-type), Syn (α-Syn transgenic), PME (PME-1 overexpressing), PME-Syn (PME-1 and α-Syn Triple transgenic), LCMT (LCMT-1 overexpressing), and LCMT-Syn (LCMT-1 and α-Syn Triple transgenic).

Fig. S2

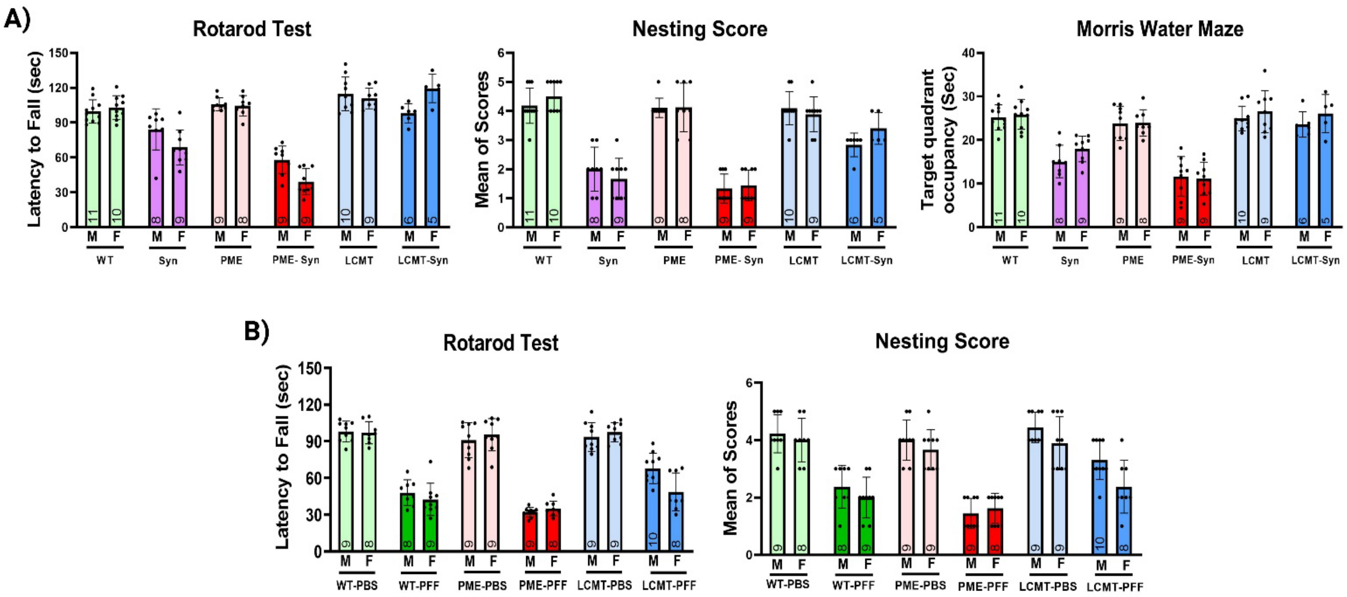

Figure S2. No significant sex differences in behavioral performance across genotypes. (A) Comparison of performance on the rotarod, nesting, and Morris Water Maze tests between male and female mice in the triple transgenic model. (B) Rotarod and nesting performance in the PFF model. No significant sex differences were observed across behavioral tests and genotypes in either model. Bar graphs show means ± SEM. Statistical analysis was performed using independent t-tests for parametric data and non-parametric t-tests where appropriate. Genotypes: WT (wild type), Syn (α-Syn transgenic), PME (PME-1 overexpressing), and LCMT (LCMT-1 overexpressing). Sample sizes (n) are indicated in the bars.

Fig. S3

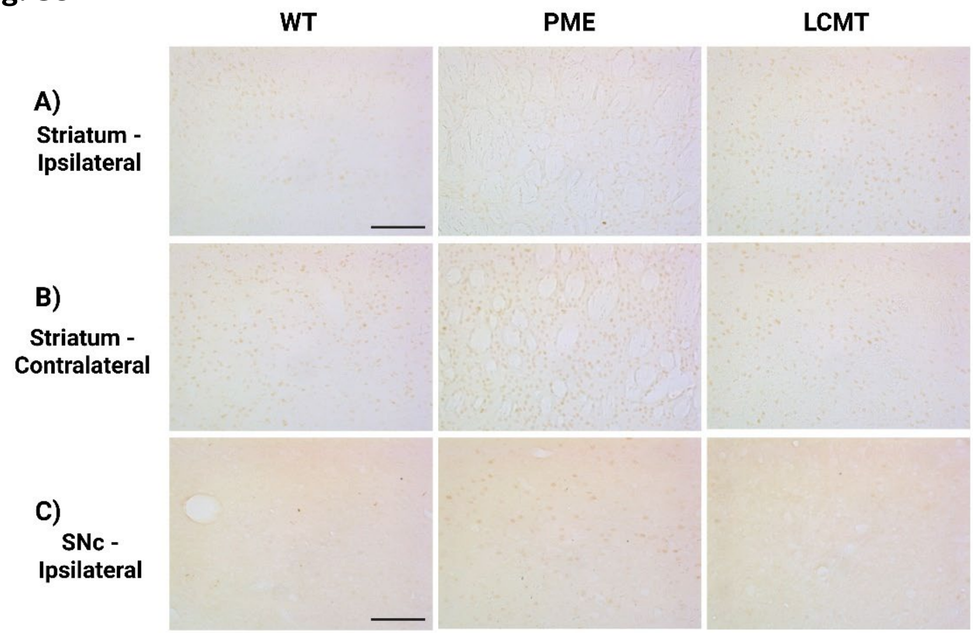

Figure S3. p-α-Syn aggregates were not detectable in brains of PBS-injected mice. Representative immunohistochemical images showing (A) p-α-Syn staining in the ipsilateral striatum, (B) contralateral striatum, and (C) ipsilateral substantia nigra. Scale bars: 100 μm. PBS-injected control mice showed no detectable accumulation of p-α-Syn aggregates in the brain. Genotypes: WT (wild-type), PME (PME-1 overexpressing), and LCMT (LCMT-1 overexpressing).
